# Distinct oscillatory mechanisms in low and high alpha-band activities for screening and potential treatment of Schizophrenia

**DOI:** 10.1038/s41398-025-03426-z

**Published:** 2025-06-23

**Authors:** Chuanliang Han, Bin Wang, Xing Peng, Meijia Li, Zhizhen Zhang, Chen Yao, Mengyu Tu, Xiaoyan Chen, Jia Zhou, Changming Wang, Xixi Zhao

**Affiliations:** 1 School of Biomedical Sciences and The Gerald Choa Neuroscience Institute, The Chinese University of Hong Kong, Hong Kong SAR, China; 2 National Clinical Research Center for Mental Disorders & National Center for Mental Disorders, Beijing Key Laboratory of Mental Disorders, Beijing Anding Hospital, Capital Medical University, Beijing, China; 3 Advanced Innovation Center for Human Brain Protection and Laboratory for Clinical Medicine, Capital Medical University, Beijing, China; 4 North China University of Technology, Tangshan, China; 5 Centre for Human Brain Health, School of Psychology, University of Birmingham, Birmingham, UK; 6 Depatment of Psychology and Center for Neuroscience, Vrije University Brussels, Brussels, Belgium; 7 Department of Mathematics and Statistics, University of Massachusetts at Amherst, Amherst, MA USA; 8 Department of Neurosurgery, The National Key Clinic Specialty, Shenzhen Key Laboratory of Neurosurgery, the First Affiliated Hospital of Shenzhen University, Shenzhen Second People’s Hospital, Shenzhen, China; 9 Zanvyl Krieger Mind-Brain Institute & Department of Psychological and Brain Sciences, Johns Hopkins University, Baltimore, MD USA; 10 School of Psychology, Fujian Normal University, Fuzhou, China; 11 Beijing Xuanwu Hospital, Capital Medical University, Beijing, China

**Keywords:** Schizophrenia, Diagnostic markers

## Abstract

Schizophrenia (ScZ) is characterized by prominent perceptual abnormalities. A deeper understanding of the neural mechanisms underlying these abnormalities is crucial for developing precise treatment strategies. Our study aimed to address the following primary questions. First, the functional role of various sub-oscillations within the alpha band remains unclear. Second, we aimed to identify biomarkers for the diagnostic purposes of ScZ. Third, the broader question of whether the diagnostic biomarker can also function as a treatment biomarker remains unknown. Resting-state EEG data from 55 ScZ patients and 61 healthy controls were analyzed to compare different sub-oscillations in the alpha band and their correlation with clinical symptoms (as measured by the general psychopathology scale). We discovered that distinct topographic patterns in low (~8 Hz) and high (~12 Hz) alpha may serve specific diagnostic and evaluative purposes respectively. Moreover, a pronounced gender bias was also observed. Low-alpha-band activity appeared to have more diagnostic relevance in females. On the other hand, the high-alpha difference was more relevant for evaluating the severity of symptoms in ScZ males. Our research has brought new insights into the neural oscillation mechanism of schizophrenia, which could substantially assist the formulating diagnosis of ScZ and the development of its treatment strategies.

## Introduction

Schizophrenia is a severe mental disorder characterized by a chronic course, marked impairments in social and vocational functioning, and a generally poor prognosis, often imposing a heavy burden on families and society [1, 2]. Long-term follow-up studies indicate that approximately 35.5% of patients experience a good or better treatment outcome following treatment, while about 40.3% face an unfavorable prognosis with significant functional impairments [3]. Moreover, the life expectancy of individuals with schizophrenia is reduced by 10–20 years [4]. As of 2019, schizophrenia still accounted for 12.2% of the Disability Adjusted Life Years (DALYs) attributed to mental disorders [5]. Despite ongoing research, the etiology and pathological mechanism underlying schizophrenia remain unclear. The diagnosis and treatment of schizophrenia are disrupted by the lack of reliable and effective biomarkers which prevents patients from having an objective and personalized diagnostic and therapeutic recommendations. There is accumulating evidence suggests that individuals with schizophrenia exhibit prominent perceptual/cognitive defects [6], particularly in the integration of information [7–9]. The brain necessitates a ‘binding mechanism’ to effectively organize and integrate information across different cortical regions into a coherent perceptual experience [10]. This mechanism, especially critical during changes in stimulus, is essential for clustering neurons and forming an ordered discharge pattern [11, 12]. Dysfunction in the discharge mechanism may contribute significantly to impairments in reality monitoring observed in schizophrenia patients [13, 14].

The impaired rhythmic activity may underpin the emergence and development of coherent cognitive and behavioral deficits, which, in turn, manifest as characteristic symptoms of psychosis and cognitive impairment [15–22]. Previous studies have elucidated that gamma oscillations carry different information and serve various neurophysiological functions [23–26]. Existing evidence suggests that gamma oscillations may play a crucial role in distinguishing ScZ during resting state [27] or feature-binding tasks [15, 28–34]. Both visually and auditory induced Gamma oscillations are important biological markers [35, 36] for diagnosing schizophrenia [31, 37–39]. It is worth noting that obtaining gamma oscillations from resting-state EEG presents certain challenges, as these, can be affected by eye movement artifacts [40] and are challenging to induce [41]. Previous research has mostly predominantly utilized MEG to induce more stable gamma oscillations [42, 43], but comes with a higher cost. In contrast, EEG provides a low-cost and convenient option for experiments involving large populations, yet current findings from EEG studies remain markedly inconsistent [44–47], in individuals with schizophrenia, it appears to be a frequent observation that there is a modest decrease alpha power in parieto-occipital, frontal and central regions when contrasted with healthy subjects [48–52]. While some studies described no reduction in terms of posterior alpha activity in schizophrenia patients [53–55]. Alpha oscillation is the most prominent feature in EEG [56, 57], yet, the relationship between alpha oscillations and ScZ is currently not well understood, potentially due to their complex nature. Most studies to date have provided only a relatively coarse measurement of power within the alpha frequency band [58–61], and variations in the defined ranges of frequency bands across different studies may contribute to discrepancies in findings. Another issue is that previous results have largely been derived from task-based studies [8, 62, 63], and complex tasks can augment the complexity of participants’ behavior. Therefore, exploring whether alpha oscillations under a simple experimental paradigm (such as resting state) and employing a simple experimental setup (such as EEG) [64], serve as a viable screening biomarker and its relationship with symptom scales is warranted.

Hence, in this study, we conducted a focused investigation into the variations of alpha oscillations to precisely characterize the differences between ScZ patients and healthy controls. Additionally, we aimed to explore the relationship between distinct alpha oscillations and the clinical symptoms. Ultimately, to enhance diagnosis and treatment strategies, we also explored the potential role of gender as a factor influencing the differentiation between ScZ and control groups, as well as its association with symptomatology.

## Materials and methods

### Participants

A total of 116 individuals participated in this study (Table 1). All participants were right-handed, possessed an educational level above elementary school, and were capable of comprehending the questionnaire content. Fifty-five patients with schizophrenia (age = 30.1 ± 6.9, 27 males 28 females) were recruited from the outpatient and inpatient department of Beijing Anding Hospital. All patients met the diagnostic criteria for schizophrenia in accordance with the Diagnostic and Statistical Manual of Mental Disorders, Fifth Edition (DSM-5). Exclusion criteria for the patients included a history of alcohol or drug abuse, severe neurological disorders (such as craniocerebral trauma, infections, or tumors), and the recent receipt of electroconvulsive therapy (ECT) or related physical therapy within the preceding 6 months. Sixty-one healthy controls (age = 28.7 ± 7.1, 30 males 31 females) were recruited through online platforms. All healthy controls underwent the MINI7.0.2 interview and were found to be free from any psychiatric diagnosis. Additionally, they had no family history of psychiatric disorders. This study was approved by the Ethics Committee of Beijing Anding Hospital, Capital Medical University (Ethics review acceptance number: 201723FS-2). All subjects provided written informed consent prior to participation.

**Table 1:** Demographic information for SCH and control groups.

Items	SCH n = 55	Control n = 61	X2/t	Pval
Age	30.1 ± 6.9	28.7 ± 7.1	−1.06	ns
Gender	27 M 28 F	30 M 31 F	<0.01	ns
PANSS total	±	na	na	na
PANSS positive	±	na	na	na
PANSS negative	±	na	na	na
PANSS general	±	na	na	na

### Assessments and procedures

We collected essential demographic information from patients via a self-compiled questionnaire, including variables such as gender and age. The severity of schizophrenia symptoms was evaluated using the Positive and Negative Syndrome Scale (PANSS), a standardized assessment tool. The PANSS is composed of 33 items, which includes 30 primary items and 3 additional supplemental items designed to assess the risk of aggression. These primary items are further subdivided into three scales: the Positive Scale, comprising 7 items; the Negative Scale, also comprising 7 items; and the General Psychopathology Scale, which includes 16 items. Each item on the subscales is rated on a scale from 1 to 7, where 1 indicates absence and 7 indicates extreme manifestations. The overall PANSS score is the aggregate of these subscale scores. The Positive Scale primarily assesses symptoms that represent an excess or distortion of normal functions. Conversely, the Negative Scale evaluates features that are diminished or absent relative to normal functioning. The General Psychopathology Scale predominantly assesses the overall severity of schizophrenic disorders.

### Electroencephalogram recordings

EEG recordings were obtained using the Net Station EEG systems equipped qwith a 128-channel EEG net from Electrical Geodesic Inc. (EGI). Participants were instructed to sit comfortably in a quiet environment devoid of electromagnetic interference. During the open-eye session, participants were required to fixate on a black cross for 5 min. This was followed by a 5-min closed-eye recording, during which participants were instructed to keep their eyes closed. Scalp EEG data were recorded at a sampling rate of 1000 Hz, with all electrode impedances maintained below 5 kΩ. Initially, the data were referenced to electrode CZ.

### Preprocess of EEG

All the data were analyzed offline utilizing Matlab (R2021b; The Math works, MA, USA) and the EEGLAB toolbox [65]. Initially, the channel sequence of data was corrected through EEGLAB to ensure a standardize data collection across all participants. The data was filtered within a frequency range of 0.1 and 30 Hz. Subsequent visual inspections were conducted, and any problematic channels/segments were removed. Independent Component Analysis (ICA) was employed to eliminate artifacts [65] (e.g., persistent muscle activity, eye blinks, and lateral eye movements). Finally, channels that were removed during earlier analysis were reinstated using spherical interpolation, and the data were re-referenced to the average of all electrodes.

### Power spectrum analysis

To accurately record alpha oscillations in EEG, we utilized the Chronux software package within the Matlab environment for spectral analysis of the collected electrophysiological data. This analysis facilitated the detailed spectrum characterization of the electrophysiological signals [66]. Chronux, an open-source software package designed for the analysis of neural data, provides comprehensive resources including current and past releases, source code, documentation, tutorials, and related publications on its homepage (http://chronux.org/). For the spectral analysis of resting EEG, a typical parameter configuration was employed (time-bandwidth product, 3; tapers, 5). This methodological approach aligns with those used in several prior studies in the field of biomedical research [67–71].

### Statistical analysis

The paired T-test was used to assess differences in the power of the 8–12 Hz alpha bands activity between open-eye and closed-eye states within both SCH and HC groups. To pinpoint significant frequency ranges, an independent sample T-test was applied to each electrode for precise alpha band activities (from 7 Hz to 13 Hz, 0.1 Hz interval) each electrode between the SCH and HC groups. Subsequently, the power at 8 Hz was fixed, and a two-way ANOVA was conducted with electrodes and population groups as factors. Furthermore, we fixed the significant brain regions to lateral parietal lobe, and a two-way ANOVA (two factors are population and gender) was conducted as shown in Fig. 4, followed by pairwise comparison using a T-test with FDR correction. Pearson correlation analysis was also utilized to examine the relationship between SCH symptoms and alpha band activities, both in the total population and when stratified by genders.

## Results

### Comparison of power across multiple alpha bands between closed-eye and open-eye states

**Fig. 1:**
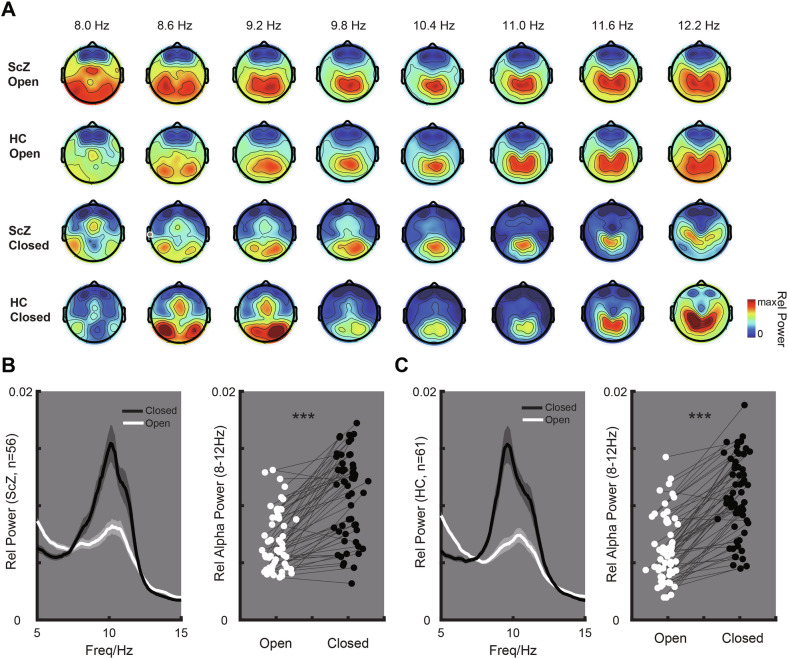
Comparison of Power Across Multiple Alpha Bands Between Closed-Eye and Open-Eye States. **A** Topographic map illustrating multiple alpha bands between closed-eye and open-eye states in both ScZ and HC groups. **B** Power spectrums in open and closed-eye states in ScZ group, and the comparison of alpha power between closed-eye and open-eye states in the ScZ group. *** is for p < 0.001. **C** Power spectrums in the open and closed-eye states in HC group, and the comparison of alpha power between closed-eye and open-eye states in the HC group. *** is for p < 0.001.

EEG field map analysis revealed distinct patterns of alpha oscillations between ScZ patients and healthy controls. During the open-eye state, the schizophrenia group exhibited pronounced low alpha (~8 Hz) oscillations extending from the central to the parieto-occipital lobe, alongside notable high alpha (~12 Hz) oscillations were observed in the central and parietal lobes. In the closed-eye state, low alpha oscillations were predominantly observed in the central to the occipital lobe, while high alpha oscillations concentrated in the central lobe. In contrast, the healthy control group during the open-eye state exhibited both high and low alpha oscillations were detected in the central and parietal lobes during the eye-open state. During the closed-eye state for the control group, low alpha oscillations were primarily identified from the central to the occipital lobe, and high alpha oscillations were prominent in the parietal lobe (Fig. 1A). Subsequent analysis compared the power across multiple alpha bands between the closed-eye and open-eye states separately for schizophrenia patients and healthy control groups. The results indicated that the relative power of the alpha band (8–12 Hz) was stronger in the closed-eye state compared to the open-eye state in both schizophrenia group (p < 0.001; Fig. 1B) and healthy control group (p < 0.001; Fig. 1C).

### Comparison of power across multiple alpha bands in schizophrenia and healthy controls

**Fig. 2:**
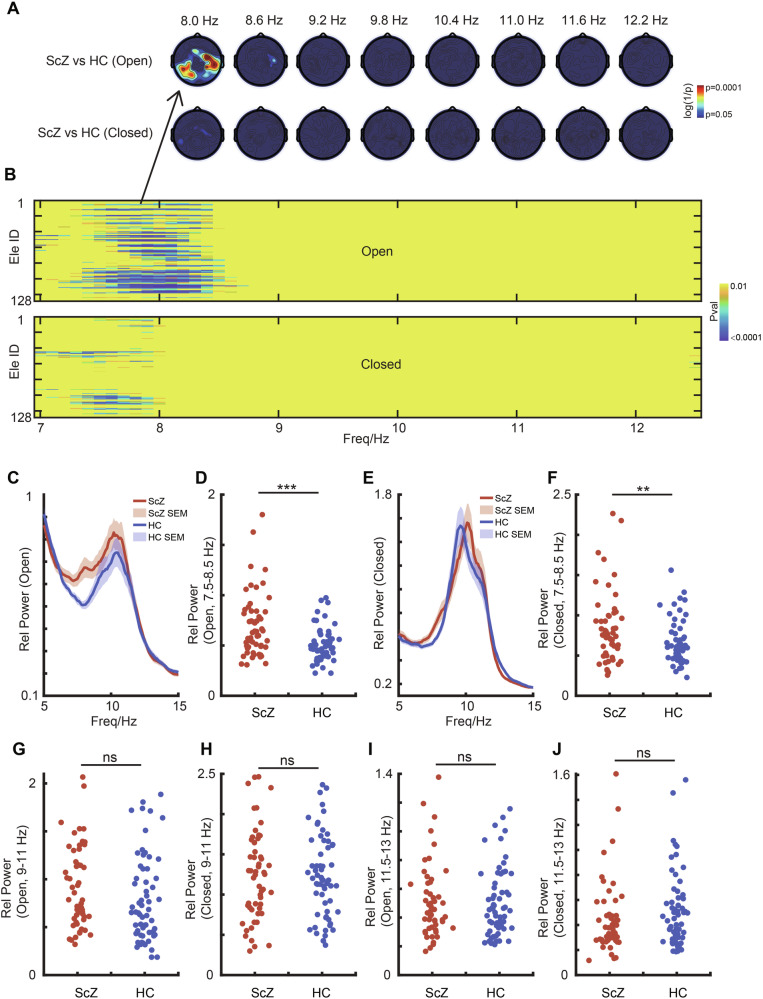
Comparison of Power Across Multiple Alpha Bands Between Schizophrenia and Healthy Control Groups. **A** Comparison of relative power in alpha band between the ScZ and HC groups in both open and closed-eye states, which is shown in the topographic maps. **B** Comparison of alpha sub-band power across all 128 electrodes in both ScZ and HC groups. The upper panel corresponds to the open-eye state, while the lower panel represents the closed-eye state. **C** Power spectrums in open-eye state in ScZ and HC group. **D** Comparison between ScZ and HC groups in low alpha band in open-eye state. Each dot represents a subject. **E** Power spectrums in closed-eye state in ScZ and HC group. **F** Comparison between ScZ and HC groups in low alpha band in closed-eye state. **G** Comparison between ScZ and HC groups in medium alpha band in open-eye state. **H** Comparison between ScZ and HC groups in medium alpha band in closed-eye state. **I** Comparison between ScZ and HC groups in high alpha band in open-eye state. **J** Comparison between ScZ and HC groups in high alpha band in closed-eye state.

To investigate the variations in alpha oscillations across different sub-bands between individuals with schizophrenia and healthy controls, several independent t-tests were conducted. The analyses indicated that individuals with schizophrenia exhibited a significantly higher power in the low alpha band (~8 Hz) compared to healthy controls in the open-eye state (p < 0.001; Fig. 2A–D). This difference was particularly notable in the temporal-occipital lobe (Fig. 2A). To further delineate these findings, a comprehensive comparison of power across all 128 electrodes in both groups was conducted, revealing notable disparities, especially in the open-eye state, predominantly in that area (Fig. 2B). However, differences in other alpha bands in the open-eye state or alpha band power during the closed-eye state between the two groups are less significant (Fig. 2E, G–J).

### The correlation between relative alpha band power and clinical symptoms in schizophrenia

**Fig. 3:**
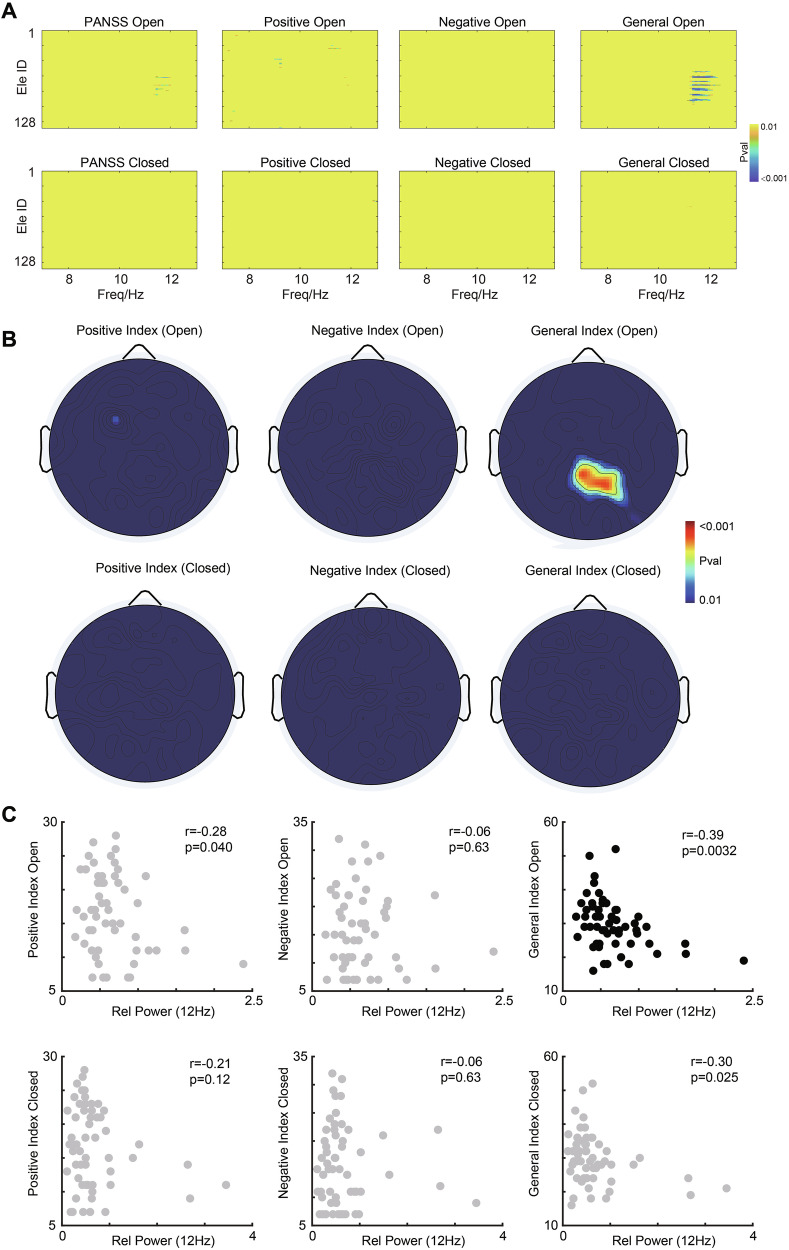
Correlation Between Clinical Symptoms and Relative Alpha Band Power in Schizophrenia Patients. **A** Correlation analysis of clinical symptoms and alpha oscillations in different alpha sub-bands. The p values are shown with colors. **B** Topographic map illustrating the significant correlation between high alpha power and positive, negative and the general psychopathology scale in the open-eye and closed-eye state. **C** Scatter plot between high alpha power and positive, negative and the general psychopathology scale in the open-eye and closed-eye state.

Subsequently, we shifted to explore the correlation between clinical symptoms in schizophrenia patients and alpha-band activities, assessing with PANSS (Fig. 3). Specifically, we investigated the relationship between the three PANSS subscales (positive, negative, and general psychopathology scale) and alpha-band activities during both open- and closed-eye states, exclusively within the schizophrenia patient cohort. The results revealed a significant negative correlation between clinical symptoms, particularly the General Psychopathology scale, and high alpha frequency (12 Hz), indicating that the higher the power was associated with, the lower the score (r = −0.38, p = 0.004; Fig. 3A–C). This correlation was most pronounced in the parietal lobe (Fig. 3B). This suggests that enhanced alpha oscillation in the parietal lobe is correlated with less severe symptoms. However, no significant correlations were observed between other alpha band powers and clinical symptoms (p > 0.05; Fig. 3A).

### Gender bias for discrimination of ScZ and control groups, and symptom correlation in ScZ group

**Fig. 4:**
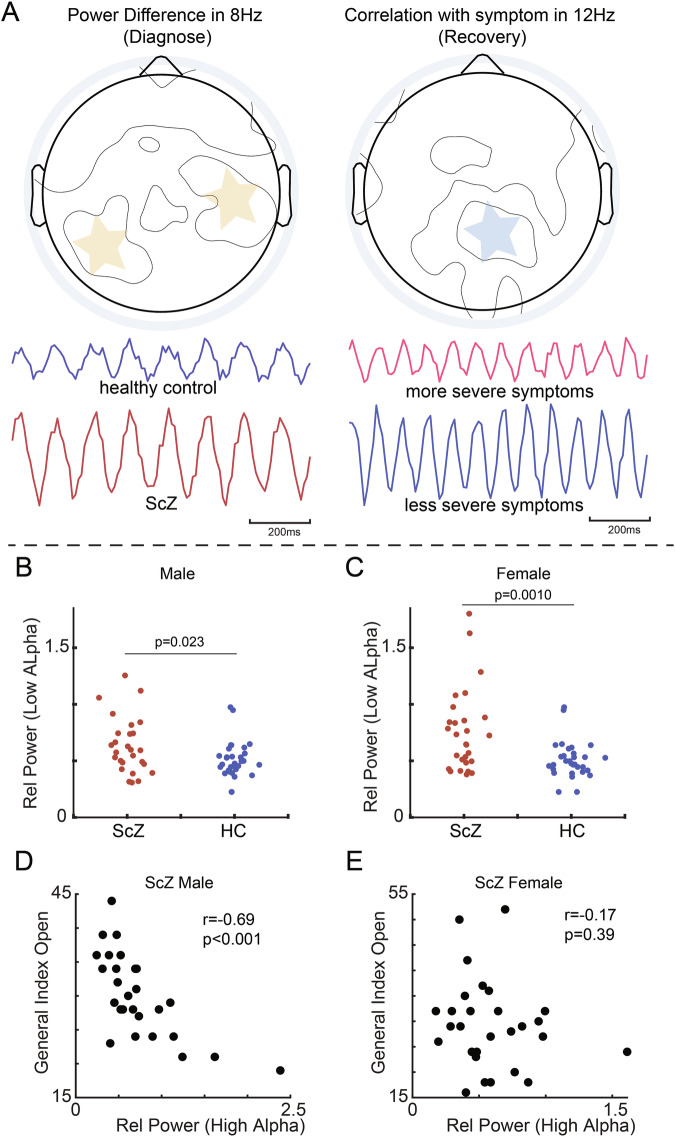
A schematic illustration of our main findings regarding the properties of low and high alpha rhythms and their gender bias. **A** A summary of findings in Fig. 2 and Fig. 3. **B** Comparison between male ScZ and male HC groups in low alpha band in open-eye state. Each dot represents a subject. **C** Comparison between male ScZ and female HC groups in low alpha band in open-eye state. Each dot represents a subject. *** is for p < 0.001. **D** Scatter plot between high alpha power and general psychopathology scale in the open-eye in male ScZ. **E** Scatter plot between high alpha power and general psychopathology scale in the open-eye in female ScZ.

Based on the main findings delineated in Figs. 2 and 3 (Fig. 4A), we extended our analysis to investigate potential gender difference effecting the power variations in low alpha band activity in the parietal-temporal lobe and correlation with symptoms in high alpha band activity in the parietal-occipital lobe. A two-way ANOVA (one factor is population; another factor is gender) was conducted with fixed brain region and frequency band (Fig. 4B–E). The results indicated a mild significance in power differences in low alpha among males (p = 0.023), but a more pronounced significance among females (p = 0.001). Moreover, a strong negative correlation was observed between high alpha power and symptoms in the parietal-occipital lobe in ScZ males (r = −0.69, p < 0.001). Conversely, this correlation was not significant in ScZ females (r = −0.17, p = 0.39).

## Discussion

Our research revealed that patients with schizophrenia exhibited significantly enhanced low alpha-band (8 Hz) activity compared to healthy controls; however, this activity was not associated with clinical symptoms. Conversely, the strength of high-alpha band (12 Hz) activity exhibited a negatively correlation with clinical symptoms. Incorporating an analysis of gender bias, these results suggest that low alpha may better represent the spectral EEG abnormalities associated with female patients with schizophrenia, thus potentially serving as a biomarker for disease classification. On the other hand, high alpha may serve as a marker for the severity of the clinical symptoms in male patients.

### Oscillatory mechanism of schizophrenia

The relative alpha power (8–12 Hz) was found to be stronger in the closed-eye state compared to the open-eye state for both schizophrenia and healthy control groups. This observation aligns with prior which demonstrates that the amplitude of alpha typically increases during eye-closed or relaxed awake states [72–74]. Specifically, in a relaxed awake state with the eyes closed, 8–12 Hz neural oscillations are the dominant rhythm, particularly, in the parietal-occipital regions [75]. This observation suggests a consistent modulation of alpha oscillations across different states of visual input, irrespective of schizophrenia status. Further analyses comparing power differences across multiple alpha bands revealed that individuals with schizophrenia exhibited significantly higher low alpha (~8 Hz) power during the open-eye state, particularly in the temporal-parietal lobe. This distinct pattern emphasizes the potential of low alpha power as a biomarker for distinguishing individuals with schizophrenia from healthy controls. Moreover, our study explored the correlation between clinical symptoms in schizophrenia patients and alpha power. We observed that high alpha (~12 Hz) power in the parietal-occipital lobe was correlated with symptoms (general psychopathology scale). This association suggests a potential link between neural activity in specific brain regions and the severity of clinical manifestations, potentially serving as a compensatory mechanism [76]. The result does not imply that low alpha and high alpha are in conflict; rather, it highlights that they are driven by distinct oscillatory mechanisms. The idea of separate neural mechanisms for these sub-oscillations has also been supported by previous studies in gamma band [24, 25, 41, 77]. Previous studies on gamma subcomponents have revealed that, in the visual system [23–25, 41, 77, 78], different gamma subcomponents are associated with distinct visual functions, such as encoding different spatial frequencies [25], orientations [79], size [24], or plaids [77], and originate from different brain regions. High gamma originates from subcortical nuclei [25, 80], medium gamma arises from the local circuits of the visual cortex, while low gamma is generated by long-range horizontal connections in the visual cortex [24]. In the hippocampal-entorhinal system [81, 82], different gamma components are also linked to different learning functions (spatial and object learning), and they originate from distinct hippocampal-entorhinal circuits. While the mechanisms of different subcomponents in the alpha band remain inconclusive, we hypothesize that, similar to the gamma band, the existence of different subcomponents implies specific functional significance, particularly regarding their respective circuits and corresponding functions. Based on existing experimental results and previous work, many studies have demonstrated that different alpha subcomponents are associated with distinct functions [71, 83–86]. Due to their different spatial distributions in EEG [70, 87], they are also likely to have different neural origin mechanisms. Future intracranial experiments need to be designed to test and verify this hypothesis.

At present, there is no unified conclusion regarding the functional differences of various types of alphas. Current research is still at the stage of describing phenomena based on data obtained under different experimental conditions [88, 89]. Early studies by predecessors suggested that different alpha sub-oscillations might be related to states [90]. High alpha appears mainly as a component of the waking EEG, middle alpha shows a difference between REM and other states, and low alpha seems to be independent of arousal states. Another study on attention also found associations with different alpha sub-oscillations [83]. The high alpha showed an inhibitory function in attention and was correlated with attentional behavioral performance. In contrast, the low alpha displayed relatively earlier activation for attended stimuli compared to unattended stimuli. A recent study on depression [71] indicated that, compared with healthy controls, depression patients had a significant increase in high alpha and a significant decrease in low alpha. This suggests that different alpha sub-oscillations also have different functional roles in mental disorders, which is exactly consistent with the conclusion of this study. We also found similar results regarding the different functions of alpha sub-oscillations in schizophrenia. However, for a more in-depth explanation, more experiments are needed in the future for further exploration.

### Neural circuit mechanism of schizophrenia

We observed a significant difference in low alpha-band activity between the ScZ group and the control group in the lateral temporal-parietal regions. Furthermore, there is a significant negative correlation between the general psychopathology scale of ScZ and high alpha power in the parietal-occipital region. These findings collectively suggest distinct neural circuit mechanisms involved in both the diagnosis and potential recovery pathways of schizophrenia. Due to the fact that EEG predominantly measures neural signals near the scalp, it may reflect underlying neural sources that cannot be precisely identified through EEG methods alone. In rodent experiments, various neural circuits are believed to be associated with schizophrenia-like symptoms [91], elucidating neural circuit mechanisms behind various underlying origins regarding the neural circuit mechanisms related to their underlying origins [92–96]. At the same time, studies at the receptor-level [30, 34, 97–99] and genetic-level [42, 100, 101] mechanisms are also crucial in regulating these abnormal sperm-differentiation-related behaviors, and the association between genes and abnormal neural oscillations has remained unclear and demands further research.

### Potential applications and future studies

Our study holds significant implications for both diagnostic and therapeutic strategies in individuals with schizophrenia. Notably, the observed gender differences underscore the necessity for clinicians to adopt gender-specific approaches in both diagnosis and treatment, which is important for optimizing clinical outcomes. However, while our results are promising, further intervention studies are essential to substantiate these conclusions robustly. Looking ahead, integrating the measurement of 8 Hz activity into standard diagnostic procedures could potentially refine the accuracy of schizophrenia diagnosis. For low alpha, although we have not yet seen a correlation between it and symptoms, the current analysis methods are relatively simple. Perhaps they have some kind of connection in the high space, which needs further exploration in the future. For therapeutic interventions, we could explore the use of Transcranial Magnetic Stimulation (TMS) or electrical stimulation methods [102] to augment high alpha power in the parietal-occipital lobe. Our next step is to collect a cohort of patients with follow-up tracking to validate the findings of this study and further emphasize the necessity of distinguishing between these sub-oscillations. Such approaches could lead to more targeted and effective treatment modalities for schizophrenia. Another limitation is we used only resting-state as an experimental condition, which is insufficient for drawing many conclusions related to cognitive processes. In the future we will combine resting state and task-based EEG together to get a clearer picture for the precise oscillatory mechanisms of schizophrenia.

## Data Availability

The dataset and are available upon reasonable request to the corresponding author.
